# An environmentally relevant concentration of antibiotics impairs the immune system of zebrafish (*Danio rerio*) and increases susceptibility to virus infection

**DOI:** 10.3389/fimmu.2022.1100092

**Published:** 2023-01-12

**Authors:** Patricia Pereiro, Magalí Rey-Campos, Antonio Figueras, Beatriz Novoa

**Affiliations:** Immunology and Genomics Group, Institute of Marine Research (IIM-CSIC), Vigo, Spain

**Keywords:** antibiotics, pollution, zebrafish, immunity, complement pathway, metatranscriptomics

## Abstract

In this work, we analysed the transcriptome and metatranscriptome profiles of zebrafish exposed to an environmental concentration of the two antibiotics most frequently detected in European inland surface water, sulfamethoxazole (SMX) and clarithromycin (CLA). We found that those animals exposed to antibiotics (SMX+CLA) for two weeks showed a higher bacterial load in both the intestine and kidney; however, significant differences in the relative abundance of certain bacterial classes were found only in the intestine, which also showed an altered fungal profile. RNA-Seq analysis revealed that the complement/coagulation system is likely the most altered immune mechanism, although not the only one, in the intestine of fish exposed to antibiotics, with numerous genes inhibited compared to the control fish. On the other hand, the effect of SMX+CLA in the kidney was more modest, and an evident impact on the immune system was not observed. However, infection of both groups with spring viremia of carp virus (SVCV) revealed a completely different response to the virus and an inability of the fish exposed to antibiotics to respond with an increase in the transcription of complement-related genes, a process that was highly activated in the kidney of the untreated zebrafish after SVCV challenge. Together with the higher susceptibility to SVCV of zebrafish treated with SMX+CLA, this suggests that complement system impairment is one of the most important mechanisms involved in antibiotic-mediated immunosuppression. We also observed that zebrafish larvae exposed to SMX+CLA for 7 days showed a lower number of macrophages and neutrophils.

## 1 Introduction

Antibiotics are chemotherapeutic agents used to impair the growth of microorganisms. According to Kümmerer et al., more than 250 different chemical substances are registered as antibiotics for agricultural, human and/or veterinary health care (1). A recent study conducted in 204 countries revealed that between 2000 and 2018, the global antibiotic consumption rate for medical purposes increased by 46%, mainly due to the increasing consumption in low- and middle-income countries (2). Together with the massive use of antibiotics due to intensive agronomy and animal farming (3, 4), this has led to a worrisome environmental situation.

In recent years, the emergence of antibiotic-resistant bacteria has attracted much attention and concern due to the impacts on global public health. The acquisition of tolerance to antibiotics is a natural evolutionary strategy of many bacterial species to compete for resources with other microorganisms, since they produce secondary metabolites that are similar to many of the synthetic antibiotics used as pharmaceuticals (5–7). Nevertheless, exposure to anthropogenic antibiotics acts as an unprecedented selection pressure that promotes the mobilization of a variety of genes known as antibiotic resistance genes (ARGs) to mobile genetic elements and their horizontal transference to many bacteria, including pathogenic species (7–9). Consequently, the high prevalence of ARGs in microbial communities and their potential spread in the environment can significantly impact the environmental microbiota and human health (8).

However, the impact of antibiotics on human health is not only due to the gradually increasing difficulties of preventing and treating bacterial infections due to the acquisition of resistance to different antibiotics but also to alterations in the human microbiota (10). The dysbiosis induced by antibiotics could favour colonization by opportunistic pathogens, since an equilibrated microbiota has a fundamental role in protection against microbial diseases (10). Certain metabolites produced by beneficial commensal microbiota and known as bacteriocins can directly inhibit the growth of opportunistic pathogens (11). Moreover, other metabolites produced by the microbiota and derived from diet components or bile acids, such as short fatty acids, amino acid derivatives, vitamins and secondary bile acids, also influence a variety of host physiological processes, such as immunity (12) and energy metabolism (13). Taken together, this evidence underscores the importance of an undisturbed host microbiota in the protection against microbial diseases and for the host inflammatory and metabolic status, which in turn affect the susceptibility to infectious diseases.

The collateral effects of antibiotics are not restricted to a sanitary or veterinary level, since they are also an important environmental concern. The presence of antibiotics in water bodies, which are important reservoirs for these compounds, is a continuous threat to microbial diversity and ecosystem functions, wildlife fauna and aquaculture species. Therefore, antibiotic pollution is a severe environmental threat that needs to be addressed, taking into consideration the One Health perspective (14), which recognizes the high interconnection among the health of humans, animals, plants and the environment.

According to the last technical report published by the Joint Research Centre (JRC) of the European Commission about the presence of antibiotics in different water sources, the more commonly found antibiotics in European inland surface waters are sulfamethoxazole (SMX) and clarithromycin (CLA), which also showed higher mean concentration levels (0.5 ng/L−17 µg/L and 0.5 ng/L−16 µg/L, respectively) (15). Because of this, we wanted to analyse the effect of an environmentally relevant concentration of these two antibiotics in the model species zebrafish (*Danio rerio*). Knowledge of the alterations of the microbiome is fundamental to elucidate its potential impacts on host health. If the impacts of antibiotic exposure at the host transcriptome level are unraveled, we can obtain better knowledge of antibiotic toxicity, microbiome-host interactions and their potential impact on the immune system. With this objective in mind, adult zebrafish were exposed to a combination of SMX and CLA (0.01 mg/L each) for 14 days, and their resistance to the viral pathogen spring viremia of carp virus (SVCV) and their intestine and kidney transcriptome and metatranscriptome profiles were analysed in the absence or presence of infection at 24 h post-infection. The obtained data provide interesting information about the effects of antibiotic environmental pollution on the microbiota of aquatic organisms and its impact on the immune response. Interestingly, SMX+CLA also affected the number of innate immune cells in zebrafish larvae, and a certain alteration of the immune cell markers was observed in adults, which suggest an interplay between microbiota, haematopoiesis and the immune response.

## 2 Materials and methods

### 2.1 Fish and viruses

Male wild-type zebrafish (18 months old) were obtained from the Instituto de Investigaciones Marinas facilities (Vigo, Spain), where zebrafish are maintained following established protocols (16, 17). When necessary, zebrafish were euthanized or anaesthetized using tricaine methanesulfonate (MS-222).

Spring viremia of carp virus (SVCV; isolate 56/70) was propagated in fibroblast-like ZF4 cells (ATCC CRL-2050) that were maintained in DMEM (Gibco) supplemented with 2% FBS (Gibco) and 1% penicillin/streptomycin solution (Gibco) at 22°C. The viral titer in the ZF4 cells (TCID_50_/mL) was calculated according to the Reed and Muench method (18).

### 2.2 Experimental exposure of zebrafish to sulfamethoxazole and clarithromycin and infection with SVCV

Sulfamethoxazole (SMX; Sigma−Aldrich; #31737) and clarithromycin (CLA; Sigma−Aldrich; #C9742) were diluted to a concentration of 10 mg/mL in DMSO, and aliquots were stored at -20°C until use.

Male adult zebrafish were divided into two tanks containing 48 fish each in a volume of 4 L of zebrafish water. One tank was supplemented with 4 mL of SMX + 4 mL of CLA (antibiotic treatment; final concentration 0.01 mg/L of each compound: DMSO at 0.2%), and the other tank served as a control and was treated with 8 mL of DMSO (vehicle; final concentration 0.2%). Every two days, half of the water (2 L) was renewed with fresh water supplemented with 4 mL of DMSO (vehicle tank) or antibiotics (2 mL of SMX and 2 mL of CLA). Fish were maintained under these conditions for 2 weeks. After that period, half of the fish from each tank (24 individuals) were anaesthetized and intraperitoneally (i.p.) infected with 20 µL of an SVCV sublethal dose (3.2 x 10^5^ TCID_50_/mL), whereas the remaining fish were i.p. inoculated with the same volume of culture medium (DMEM + 2% FBS + penicillin/streptomycin) that was maintained in contact with ZF4 cells but in the absence of viral particles. At 24 hours post-infection (hpi), four fish from each condition (DMSO-Control, DMSO-SVCV, Antibiotics-Control and Antibiotics-SVCV) were sacrificed, and the full intestine and kidney were sampled and stored at -80°C until RNA isolation and transcriptome sequencing. The remaining fish were divided into two tanks per condition (10 fish/tank) and exposed to the corresponding treatments (DMSO or antibiotics) for mortality monitoring.

To test the effect of a lethal dose of SVCV, a total of 60 zebrafish were exposed to antibiotics or vehicle. After two weeks, half of the fish from each treatment (30 individuals) were i.p. infected with 20 µL of an SVCV lethal dose (6.4 x 10^6^ TCID_50_/mL), whereas the remaining fish were i.p. inoculated with the same volume of culture medium. The fish were divided into three tanks per condition (10 fish/tank) for mortality monitoring.

### 2.3 RNA isolation and transcriptome sequencing

Total RNA from intestine and kidney samples obtained from the fish exposed to DMSO or antibiotics was extracted using the Maxwell RSC simplyRNA Tissue kit (Promega) with an automated Maxwell^®^ RSC 48 Instrument in accordance with the manufacturer’s instructions. For the samples obtained from the sublethal infection, the quantity of RNA was measured in a Qubit 4 Fluorometer (Invitrogen) using the Qubit RNA HS Assay Kit (Invitrogen); after this, RNA integrity was analysed in an Agilent 2100 Bioanalyzer (Agilent Technologies Inc., Santa Clara, CA, USA) according to the manufacturer’s instructions. All the samples that passed the quality control tests were used for Illumina library preparation.

Double-stranded cDNA libraries were constructed using the TruSeq Stranded mRNA LT Sample (Illumina, San Diego, CA, USA). Paired-end 150 bp (PE150) sequencing was performed on an Illumina NovaSeq 6000 sequencer. Both library preparation and sequencing were performed at Macrogen Inc. (Seoul, Republic of Korea).

The raw read sequences obtained were deposited in the Sequence Read Archive (SRA) (http://www.ncbi.nlm.nih.gov/sra) under the BioProject accession number PRJNA893568.

### 2.4 Trimming, RNA-Seq and differential expression analysis

To CLC Genomics Workbench v. 21.1 (CLC Bio, Aarhus, Denmark) was used to filter and trim reads and conduct the RNA-Seq analyses against the last version of the zebrafish genome (GRCz11). Raw reads were trimmed to remove adaptor sequences and low-quality reads with a quality score limit of 0.05. RNA-Seq analyses were performed using the zebrafish genome with the following parameters: length fraction = 0.8, similarity fraction = 0.8, mismatch cost = 2, insertion cost = 3 and deletion cost = 3. Finally, a differential expression analysis test was performed with the DESeq2 package (19) in R Studio v. 4.1.3 to compare gene expression levels and identify differentially expressed genes (DEGs). A filtering step was performed to remove low-expression genes from the analysis (only genes showing 10 counts were retained). The results were corrected using the lfcShrink function of the apeglm R package (20). Genes were considered differentially expressed when they presented an absolute log2-fold change ≥ 1 and p value < 0.05.

### 2.5 Gene Ontology and KEGG pathway enrichment analyses and protein–protein interaction networks

For the DEGs between SVCV-infected and uninfected zebrafish, we conducted GO enrichment analysis of biological processes and KEGG pathway analysis using David software (21). The significance level was set at a p value ≤ 0.05 in all cases, and the minimal gene count was set at 3. The representation of the different categories was based on the fold-enrichment value.

The interactions of the proteins encoded by the DEGs of interest were analysed with STRING v11.5 software (https://string-db.org) (22).

### 2.6 Venn diagrams, heatmaps and principal component analysis

Venn diagrams were constructed with the Venny 2.1 tool (http://bioinfogp.cnb.csic.es/tools/venny/. Using the mean TPM values of the selected DEGs, heatmaps were constructed using the average linkage method with the Euclidean distance using the Clustvis web tool (23) (https://biit.cs.ut.ee/clustvis/). PCA plots were also generated with Clustvis using the TPM values.

### 2.7 Quantification of SVCV replication and validation of the RNA-Seq data by quantitative PCR

The RNA isolated from the intestine and kidney of DMSO-Control, Antibiotics-Control, DMSO-SVCV and Antibiotics-SVCV zebrafish was retrotranscribed with the NZY First-Strand cDNA Synthesis Kit (NZYtech) using 0.5 and 0.15 µg of total RNA for intestine and kidney, respectively. qPCRs were performed using specific primers designed with Primer 3 software (24) and evaluated for their specificity, and their efficiencies were tested according to the protocol described by Pfaffl (25). Individual qPCRs were conducted in 25-µl reaction volumes using 12.5 µL of SYBR GREEN PCR Master Mix (Applied Biosystems), 10.5 µL of ultrapure water (Sigma–Aldrich), 0.5 µL of each specific primer (10 µM) and 1 µL of cDNA template. All reactions were performed using technical triplicates in a 7300 Real-Time PCR System thermocycler (Applied Biosystems) with an initial denaturation step (95°C, 10 min), followed by 40 cycles of a denaturation step (95°C, 15 s) and one hybridization-elongation step (60°C, 1 min). The relative expression levels of the different genes were normalized following the Pfaffl method (25) using 18S ribosomal RNA (18S) as a reference gene. Fold change units were calculated by dividing the normalized expression values of stimulated tissues by the normalized expression values of the controls. SVCV replication was detected by the relative expression of the SVCV nucleoprotein (N) gene. For RNA-Seq validation, seven genes belonging to the complement pathway (*c1s*, *c3a.1*, *c4*, *c5*, *c8a*, *c9*, *masp1*) and two genes involved in blood coagulation (*f5*, *plg*) and haemoglobin synthesis (*cp*, *tfa*) were selected to confirm the results. Additionally, the expression of the cell markers *marco*, *mpeg1.1* and *mpx* in the samples was analysed. The primer pairs used in this work are listed in **Supplementary Table S1**.

### 2.8 Bacterial and fungal taxonomic profiling

To analyse the bacterial and fungal profiles of the sequenced reads in the experiment, a work plan was developed consisting of mapping the reads to reference databases. The host-specific reads were filtered by mapping the reads to the zebrafish genome (GRCz11). The microorganism reference databases used in this work were built from all the fungal genomes deposited in RefSeq at 18/05/2022, containing 388 assemblies. Additionally, the curated bacterial database included in the CLC Microbial Genomics Module (including 36,027 bacterial assemblies) was used to perform the analyses. CLC Microbial Genomics Module 21.1 software (Qiagen) was used to perform the taxonomic profiling.

The mapping parameters used to classify the reads into different taxonomic groups were as follows: length fraction = 0.5, similarity fraction = 0.8 and a minimum seed length of 30. The minimum seed length parameter defines the minimum perfect match length for a position in the reference to be considered a valid candidate when matching the read.

The alpha diversity was calculated using the vegan R package (26) to allow for us to describe some community ecology parameters. In addition, the Simpson’s index, Shannon entropy, Fisher’s alpha parameter and species number were evaluated. A pairwise Mann−Whitney U test was performed to determine which pairs of groups followed different distributions.

To confirm the higher bacterial load in the fish exposed to antibiotics, we conducted qPCR analysis of the 16S rRNA using the universal primer pair PSL-PSR (27) (**Supplementary Table S1**). For this, we used the same RNA samples and qPCR conditions as in the previous section.

### 2.9 Zebrafish larvae exposure to antibiotics and imaging of macrophages and neutrophils

Double-transgenic *Tg(mpeg:mCherry/mpx:GFP)* embryos (7 hours postfertilization (hpf)) in 6-well plates were exposed for 7 days to DMSO (0.2%) or SMX+CLA (0.01 mg/L each; 0.2% DMSO). The water containing the treatments was renewed every two days. From 24 hpf, the embryo larvae were also treated with 0.2 mM 1-phenyl 2-thiourea (PTU; Sigma−Aldrich) to prevent pigment formation. A total of 25 larvae per treatment (5 biological replicates/5 larvae per replicate) were sampled for qPCR analysis of macrophage (*marco*, *mpeg1.1*) and neutrophil (*mpx*) markers and stored at -80°C until use. The primer pair used is listed in **Supplementary Table S1**, and the qPCR conditions were the same as those described in Section 2.7. Another 12 larvae per treatment were anaesthetized with 0.003% MS-222 (Sigma−Aldrich) and used for microscopy analysis. Fluorescence images were taken for each experimental condition with a Leica DMi8 microscope (Leica Microsystems) equipped with GFP and TXR filters to visualize Mpx+ and Mpeg+ cells, respectively. Cells were counted with Fiji software (28).

### 2.10 Statistical analyses

Graphs and statistical analyses were performed using GraphPad Prism software 8.0.1 (GraphPad, CA, USA). Survival data were analysed with Kaplan–Meier survival curves, and statistically significant differences were determined with a log-rank (Mantel−Cox) test. Gene expression results are represented graphically as the mean ± standard error (SEM) of the biological replicates. Larval cell counts are represented as the mean ± SEM. To determine significant differences, data were analysed with Student’s t test. Significant differences are defined as *** (0.0001 < p < 0.001), ** (0.001 < p < 0.01) or * (0.01 < p < 0.05).

## 3 Results

### 3.1 Antibiotic exposure alters intestinal and kidney microbial profiles

**Figure 1:**
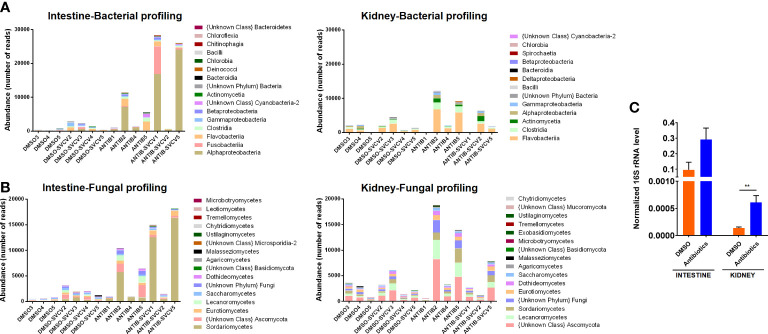
Microbiota abundance. Absolute abundance of the main **(A)** bacterial and **(B)** fungal classes in intestine and kidney samples from zebrafish exposed to SMX+CLA or the vehicle alone and infected or not with SVCV. **(C)** The total abundance of bacteria was measured in the intestine and kidney of uninfected SMX+CLA- or DMSO-treated fish by qPCR detection of the 16S rRNA using the universal primer pair PSL-PSR. Statistically significant differences are displayed as ** (p<0.01).

The bacterial and fungal profiles across the different intestine and kidney samples reveal a tendency towards a higher absolute abundance of reads from bacteria (**Figure 1A**) and fungi (**Figure 1B**) in the intestine and kidney of those fish exposed to antibiotics. The higher abundance of total bacteria was confirmed by qPCR analysis of the 16S rRNA (**Figure 1C**). As expected, the total abundance of bacteria was higher in the intestine than in the kidney (**Figure 1C**).

**Figure 2:**
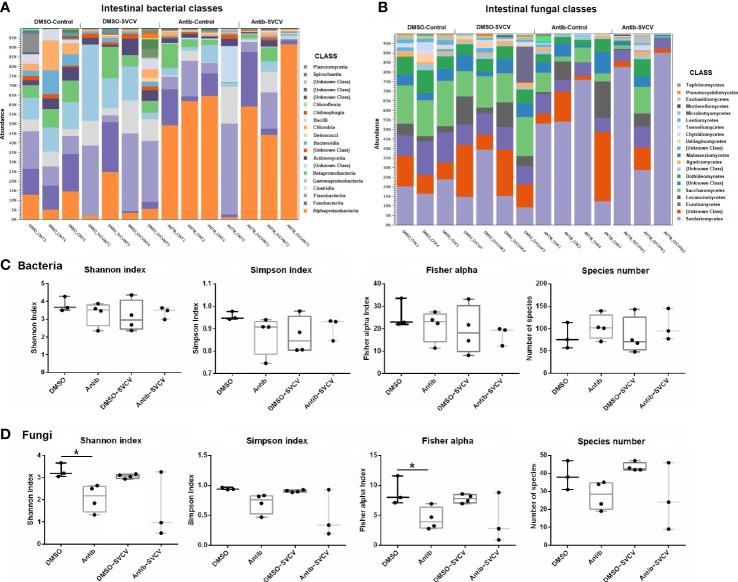
Taxa relative abundances in the intestine of zebrafish treated with DMSO (vehicle) or SMX+CLA under SVCV-infected and uninfected conditions. **(A)** Relative abundance of the main bacterial classes. **(B)** Relative abundance of the main fungal classes. **(C)** Shannon entropy, Simpson, Fisher alpha and species number indices of **(C)** bacteria and **(D)** fungi in the intestine of the experimental groups. Statistically significant differences are displayed as * (p<0.05) after the Mann−Whitney U test.

The analysis of the relative abundance showed a different pattern of bacterial (**Figure 2A**) and fungal (**Figure 2B**) classes in the intestine between fish treated with antibiotics and those exposed to the vehicle alone. In the intestines of DMSO-treated organisms, the most abundant bacterial classes were Flavobacteriia (21.7 ± 14.73%), Gammaproteobacteria (16.43 ± 11.31), Fusobacteriia (10.89 ± 9.85%), Alphaproteobacteria (9.85 ± 8.17%) and Clostridia (8.94 ± 5.08%), whereas the most abundant class in fish exposed to SMX+CLA was Alphaproteobacteria (53.18 ± 27.44%), followed by Flavobacteriia (14.72 ± 16.06%), Fusobacteriia (9.79 ± 10.56%), Clostridia (5.47 ± 7.18%) and Betaproteobacteria (4.33 ± 4.49%) (**Figure 2A**). Five bacterial classes showed a differential abundance between DMSO- and antibiotic-treated zebrafish, comprising Chloroflexia and Alphaproteobacteria, which showed a higher abundance in the intestine of zebrafish exposed to SMX+CLA, and Actinomycetia, Bacilli and Gammaproteobacteria, which showed a lower relative abundance (**Supplementary File S1**). The high abundance of Alphaproteobacteria in the intestine of antibiotic-treated fish mainly corresponded to the order Rhodospirillales.

With regard to the intestinal fungi, the most abundant classes in zebrafish exposed to DMSO alone were Sordariomycetes (19.94 ± 9.76%), Saccharomycetes (18.29 ± 3.56%), Eurotiomycetes (12.47 ± 3.01%), Dothideomycetes (7.68 ± 2.25%) and Lecanoromycetes (6.81 ± 4.97%); in fish exposed to antibiotics, the most abundant fungal classes were Sordariomycetes (56.79 ± 28.66%), Eurotiomycetes (7.6 ± 2.12%), Saccharomycetes (5.9 ± 4.87%), Lecanoromycetes (5.36 ± 7.21%) and Dothideomycetes (3.82 ± 3.15%) (**Figure 2B**). Six fungal classes showed a significantly different abundance in the intestines of DMSO- and antibiotic-treated zebrafish, which include five classes with lower relative abundance after antibiotic exposure (Dothideomycetes, Eurotiomycetes, Leotiomycetes, Saccharomycetes and Exobasidiomycetes), and one class, Sordariomycetes, with higher abundance in fish treated with SMX+CLA (**Supplementary File S2**).

No significant differences were observed in the relative abundance of bacterial and fungal classes in kidney samples from DMSO- and antibiotic-treated fish (**Supplementary Figure S1**; **Supplementary Files S1** and **S2**). The predominant classes of bacteria in the kidneys of the DMSO and antibiotic-treated fish were Flavobacteriia (44.07 ± 18.02% and 47.12 ± 15.68%, respectively), Clostridia (17.06 ± 3.82% and 17.12 ± 4.92%, respectively), Alphaproteobacteria (12.18 ± 10.62% and 5.71 ± 4.05%, respectively), Gammaproteobacteria (8.95 ± 5.65% and 6.88 ± 3%, respectively) and Actinomycetia (5.17 ± 2.24% and 8.29 ± 6.69%, respectively) (**Supplementary Figure S1**); for fungi, the predominant classes in the kidney were Sordariomycetes (12.7 ± 3.23% and 14.95 ± 4.74%, respectively), Eurotiomycetes (12.54 ± 4.64% and 9.99 ± 3.71%, respectively), Lecanoromycetes (12.06 ± 6.55% and 13.85 ± 6.1%, respectively), Saccharomycetes (8.7 ± 4.3% and 6 ± 3.16%, respectively) and Dothideomycetes (6.89 ± 1.66% and 6.21 ± 1.38, respectively) (**Supplementary Figure S1**).

The alpha diversity indices did not reveal significant differences in the bacteria in either the intestine or kidney (**Figure 2C** **;** **Supplementary Figure S1**). For the fungi, a lower alpha diversity was observed in the intestines of fish exposed to SMX+CLA, according to the Shannon and Fisher alpha diversity indices (**Figure 2D**).

### 3.2 Adult zebrafish exposed to sulfamethoxazole and clarithromycin are more susceptible to SVCV

**Figure 3:**
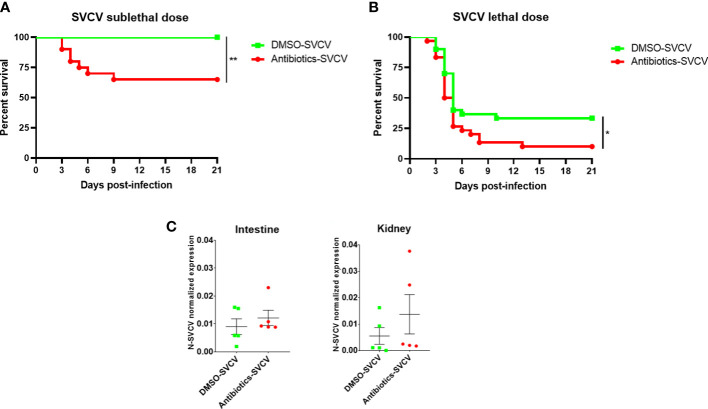
The long-term exposure of zebrafish to SMX+CLA conditions their resistance to SVCV. Kaplan−Meier survival curves of SMX+CLA- or DMSO-treated fish infected with a **(A)** sublethal or **(B)** lethal dose of SVCV. Statistically significant differences are displayed as ** (p<0.01). No mortality events were registered for the uninfected fish. **(C)** qPCR detection of the SVCV N gene in the intestine and kidney of zebrafish infected with SVCV at 24 hpi.

When adult zebrafish were exposed for 14 days to 0.01 mg/L SMX and CLA and then infected with a sublethal dose of SVCV, the fish exposed to the vehicle alone (0.2% DMSO) showed a survival rate of 100%, whereas the animals exposed to the combination of both antibiotics showed a significantly lower survival (65%) (**Figure 3A**). In addition, infection with a highly lethal dose resulted in a survival rate of 33.33% for the DMSO-SVCV group, and this percentage decreased for the Antibiotics-SVCV zebrafish to 10% (**Figure 3B**).

SVCV replication was analysed in the intestine and kidney of 5 infected fish from each group at 24 hpi and, although no statistically significant differences were observed, the fish exposed to an environmentally relevant concentration of sulfamethoxazole and clarithromycin showed a tendency towards higher SVCV replication (**Figure 3C**).

### 3.3 RNA-Seq and differential expression analysis of the intestine and kidney of zebrafish exposed to antibiotics or vehicle alone in the absence or presence of a sublethal SVCV infection.

To better elucidate the alteration of the transcriptome response due to chronic exposure to 0.01 mg/L SMX+CLA, high-throughput transcriptome sequencing and RNA-Seq analyses were conducted with uninfected and SVCV-infected fish at 24 hpi. Four individuals were sampled for each experimental condition, although only three individuals were sequenced for the DMSO-control and antibiotics-SVCV groups due to the low quantity and/or quality of the RNA.

A total of 284,485,932 raw reads were obtained from the intestine samples of the 14 individuals sequenced, with an average value of 20,320,424 reads per sample; of the total raw reads, more than 99.99% successfully passed the quality control and showed an average length of 145.01 bp. From these high-quality reads, a mean of 96.92% successfully mapped to the zebrafish genome, and 3.08% of the reads remained unmapped. For the kidney samples, a total of 281,052,036 raw reads were obtained, with an average value of 20,075,145 reads per sample; of the total reads, more than 99.99% successfully passed the quality control and showed an average length of 144.4 bp. From these high-quality reads, a mean of 96.66% successfully mapped to the zebrafish genome, and 3.35% of the reads remained unmapped. The individual sample statistics are shown in **Supplementary Table S2**.

#### 3.3.1 RNA-Seq and differential expression analysis of the intestine and kidney of zebrafish exposed to antibiotics or vehicle alone in the absence or presence of a sublethal SVCV infection

**Figure 4:**
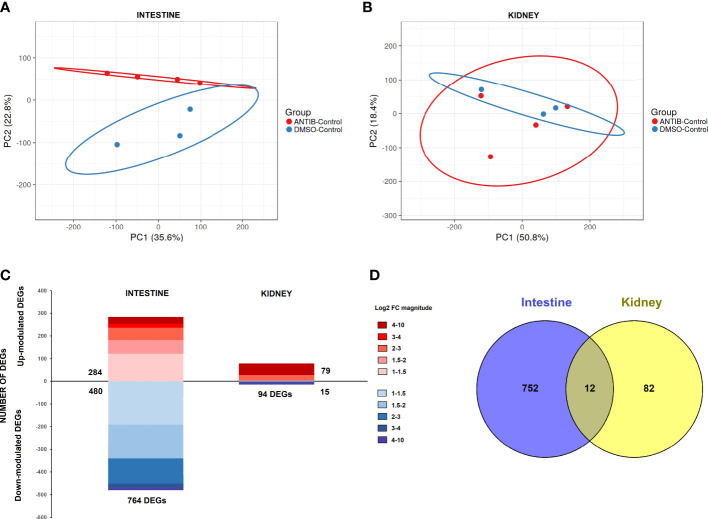
Comparative transcriptome analyses of zebrafish exposed to antibiotics or vehicle (DMSO) in the absence of infection. Principal component analyses (PCAs) of the **(A)** intestine and **(B)** kidney. **(C)** Stacked column charts reflecting the number and intensity (in log2 FC value) of the DEGs in the intestine and kidney of fish exposed to SMX+CLA for 2 weeks compared to the corresponding control (DMSO-treated fish). **(D)** Venn diagram showing the number of common and exclusive DEGs in the intestine and kidney.

Using the TPM values obtained from RNA-Seq analyses, PCA was performed to determine the sample distribution in uninfected fish, only taking into consideration the effect of the antibiotics. Whereas the PCA plot for intestine samples showed a clear differentiated distribution between DMSO- and antibiotic-treated fish (**Figure 4A**), this pattern was not observed for kidney samples, which were more randomly distributed (**Figure 4B**). This evidence suggests a stronger effect of antibiotic treatment on the intestine than on the kidney, which was corroborated by the differential expression analyses. In the intestine, the comparison of antibiotic-control vs. DMSO-control showed a total of 764 DEGs (284 upregulated and 480 downregulated), whereas with the same statistical parameters, only 94 DEGs were obtained for the kidney (79 upregulated and 15 downregulated genes) (**Figure 4C**). The full repertoire of DEGs is provided in **Supplementary File S3**. Moreover, only 12 DEGs were commonly modulated by antibiotics in the intestine and kidney (**Figure 4D**).

#### 3.3.2 Exposure to antibiotics significantly alters the response to a virus

**Figure 5:**
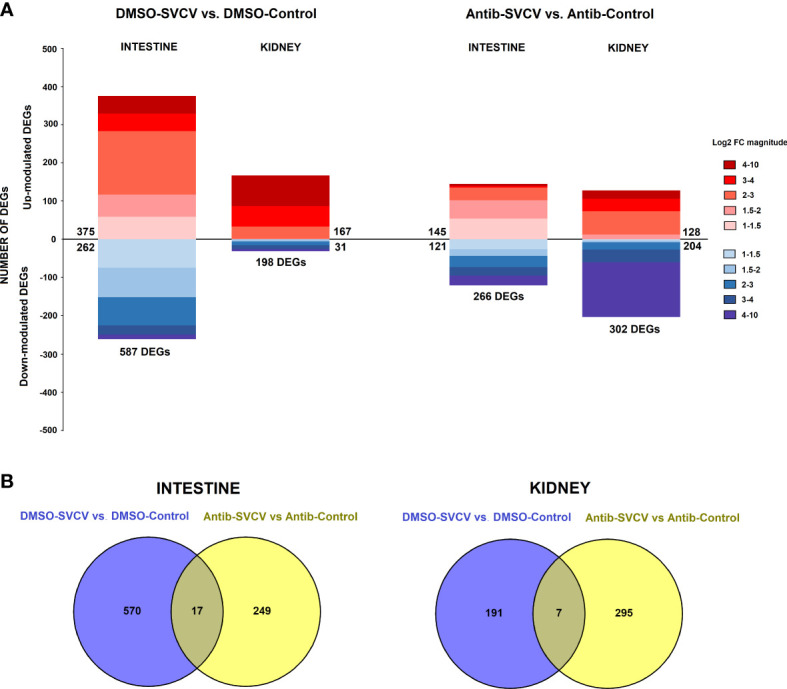
Comparative transcriptome analyses of zebrafish exposed to antibiotics or vehicle (DMSO) and infected with SVCV. **(A)** Stacked column charts reflecting the number and intensity (in log2 FC value) of the DEGs in the intestine and kidney at 24 hpi with SVCV. **(B)** Venn diagrams showing the number of common and exclusive DEGs after SVCV infection in DMSO- and SMX+CLA-treated fish.

The analysis of the response to SVCV at 24 hpi in fish exposed to SMX-CLA (antibiotics-SVCV vs. antibiotics-control) or vehicle alone (DMSO-SVCV vs. DMSO-control) revealed a completely different expression pattern. In the animals exposed to DMSO, a total of 587 DEGs were significantly modulated in the intestine (375 upregulated and 262 downregulated genes), whereas the effect of the viral challenge induced a lower response in fish exposed to antibiotics in terms of the number of DEGs, with 266 DEGs (145 upregulated and 121 downregulated genes) (**Figure 5A**). Moreover, only 17 genes were commonly modulated in the intestine for both groups (**Figure 5B**), and 5 were modulated in opposite ways. In the kidney, a higher number of DEGs, 302, was observed in zebrafish exposed to antibiotics after SVCV challenge, which were mainly downregulated (128 upregulated and 204 downregulated DEGs); this pattern was not observed for the animals exposed to DMSO, which showed 198 DEGs, with most upregulated (167 upregulated and 31 downregulated genes) (**Figure 5A**). As occurs in the intestine, a low number of DEGs were commonly modulated after SVCV infection in the kidney, with only 7 common DEGs (**Figure 5B**), two of which were modulated in opposite ways. The full repertoires of DEGs for the comparisons DMSO-SVCV vs. DMSO-control and antibiotics-SVCV vs. antibiotics-control are provided in **Supplementary File S4**, respectively.

### 3.4 GO and KEGG enrichment analyses

**Figure 6:**
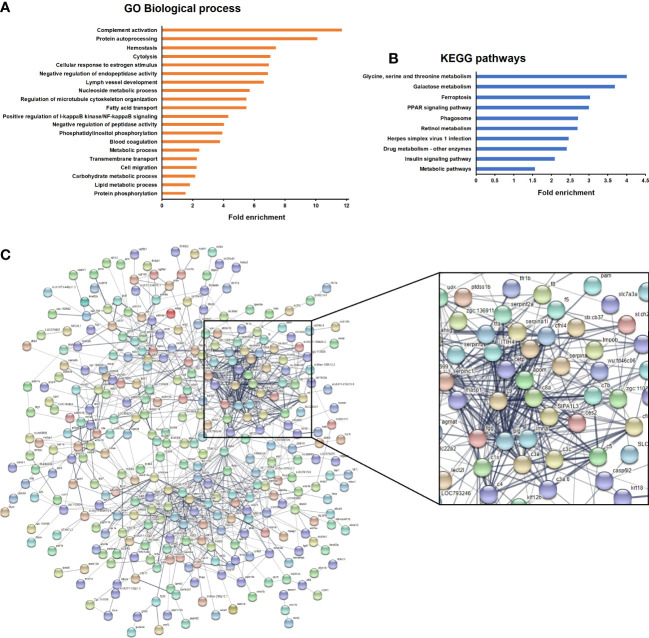
Enrichment analyses of the genes significantly modulated in the intestine of fish after exposure to antibiotics compared to the DMSO-treated group. **(A)** Significantly enriched GO biological processes. **(B)** Significantly enriched KEGG pathways. **(C)** String protein−protein interaction network showing the main gene clusters modulated by exposure to antibiotics.

GO and KEGG enrichment analyses were conducted to elucidate the main biological processes affected by the exposure to antibiotics. No biological processes (BPs) or KEGG pathways were significantly enriched for kidney with the selected filters. However, several BPs were found to be significantly enriched in the intestine, with “complement activation” being the most enriched BP (**Figure 6A**). Other processes intimately linked to the complement were also overrepresented in the data, such as cytolysis, haemostasis and blood coagulation. With regard to the KEGG pathways, the term “Herpes simplex virus 1 infection” was significantly enriched and included, among others, the DEGs belonging to the complement cascade (**Figure 6B**). This strong modulation of complement- and coagulation-related genes was also clearly reflected in a STRING protein-protein interaction network constructed with the genes modulated in the intestine after the exposure to antibiotics (**Figure 6C**). Complement and coagulation genes formed the strongest cluster of the network.

With regard to the response to SVCV in DMSO- or antibiotic-treated zebrafish, certain GO terms were significantly enriched in both the intestine (**Supplementary Figure S2**) and kidney (**Supplementary Figure S3**) in the presence or absence of antibiotics. Interestingly, the term “Complement activation” was only significantly enriched in the kidney of fish treated with the vehicle DMSO.

### 3.5 Antibiotic exposure significantly impaired the transcription of complement components in the intestine and limited their upregulation in the kidney after SVCV infection

A protein−protein interaction network analysis of the proteins encoded by the genes downregulated in intestine of fish exposed to SMX-CLA revealed that the strongest cluster of proteins was composed of a multitude of complement-, coagulation- and haemoglobin synthesis-related molecules (**Figure 6B**). This main cluster was interconnected through different integrin members (*itgb2*, *itgb1b*, *itgav* and *itgae.2*) with a secondary cluster highly enriched in genes with a role in different aspects of the immune response, including chemotaxis (*erbb2*, *ptpn11b*), B-cell maturation and activation (*syk*, *blnk*), regulation of inflammation (*socs1b*, *socs5a*), pathogen recognition receptors (*tlr5a*) and macrophage-specific antibacterial proteins (*mpeg1.1*, *mpeg1.2*), among others.

**Figure 7:**
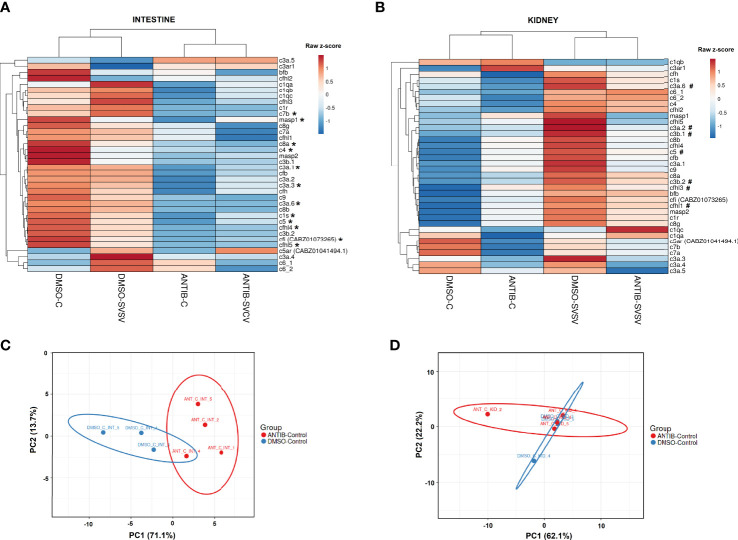
Analysis of the complement components. Heatmaps representing the mean TPM values of the different complement components in the **(A)** intestine and **(B)** kidney of the four experimental groups (DMSO-C, DMSO-SVCV, ANTIB-C and ANTIB-SVCV). * Indicates statistically significant differences between the ANTIB-C and DMSO-C groups; # indicates statistically significant differences between the DMSO-SVCV and DMSO-C groups. PCA of the complement genes in the **(C)** intestine and **(D)** kidney of the DMSO-control and antibiotics-control groups.

Since the main biological process affected by the exposure to antibiotics in the intestine was the complement pathway, and this was also one of the main processes induced in the kidney after SVCV infection in fish exposed to DMSO, but not in those fish exposed to SMX-CLA, we analysed this immune mechanism in more detail. Heatmaps representing the mean expression levels (in log2[TPM]) of the different complement components in the intestine through the different experimental treatments revealed that most of the complement components showed a lower expression in both the antibiotics-control and antibiotics-SVCV groups compared to the DMSO-control and DMSO-SVCV groups (**Figure 7A**). Twelve of these complement components (*c1s*, *c3a.1*, *c3a.3*, *c3a.6*, *c4*, *c5*, *c7b*, *c8a*, *c9*, *masp1*, *cfi*, *cfhl4*, *cfhl5*) were significantly downregulated in antibiotic-treated fish compared to DMSO-treated control fish (denoted by an asterisk *, **Figure 7A**; **Supplementary File S3**). The PCA of the complement genes in intestine samples from uninfected fish showed a good separation between the antibiotic-treated fish from those exposed to the vehicle alone (**Figure 7C**). On the other hand, whereas no significant differences were observed in the kidney for the complement components between DMSO-control and antibiotic-control (**Figures 7B, D**; **Supplementary File S3**), the heatmap reveals an overall higher expression of the complement genes in the DMSO-SVCV fish (**Figure 7B**). Seven of these genes (*c3a.2*, c3a.6, *c3b.1*, *c3b.2*, *c5*, *cfhl3*, *cfhl1*) were significantly upregulated at 24 hpi in the DMSO-SVCV group compared to the DMSO-control group (denoted by a hash #, **Figure 7B**), whereas no complement genes were modulated in those fish exposed to antibiotics after SVCV infection (**Supplementary File S4**).

**Figure 8:**
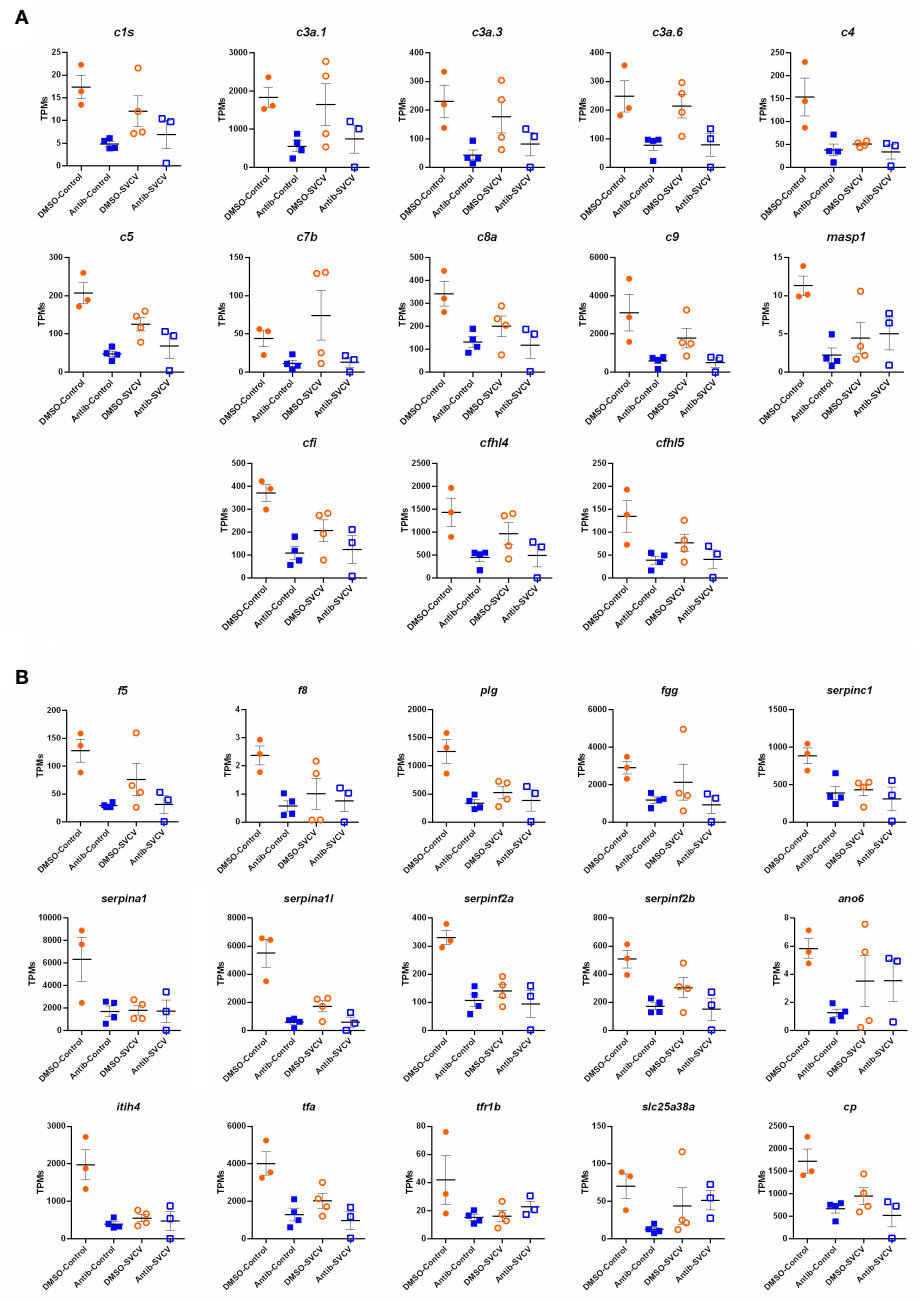
Representation of the TPM values of the **(A)** complement and **(B)** coagulation genes significantly modulated in the intestines of fish exposed to SMX-CLA compared to those exposed to DMSO (vehicle).

**Figure 9:**
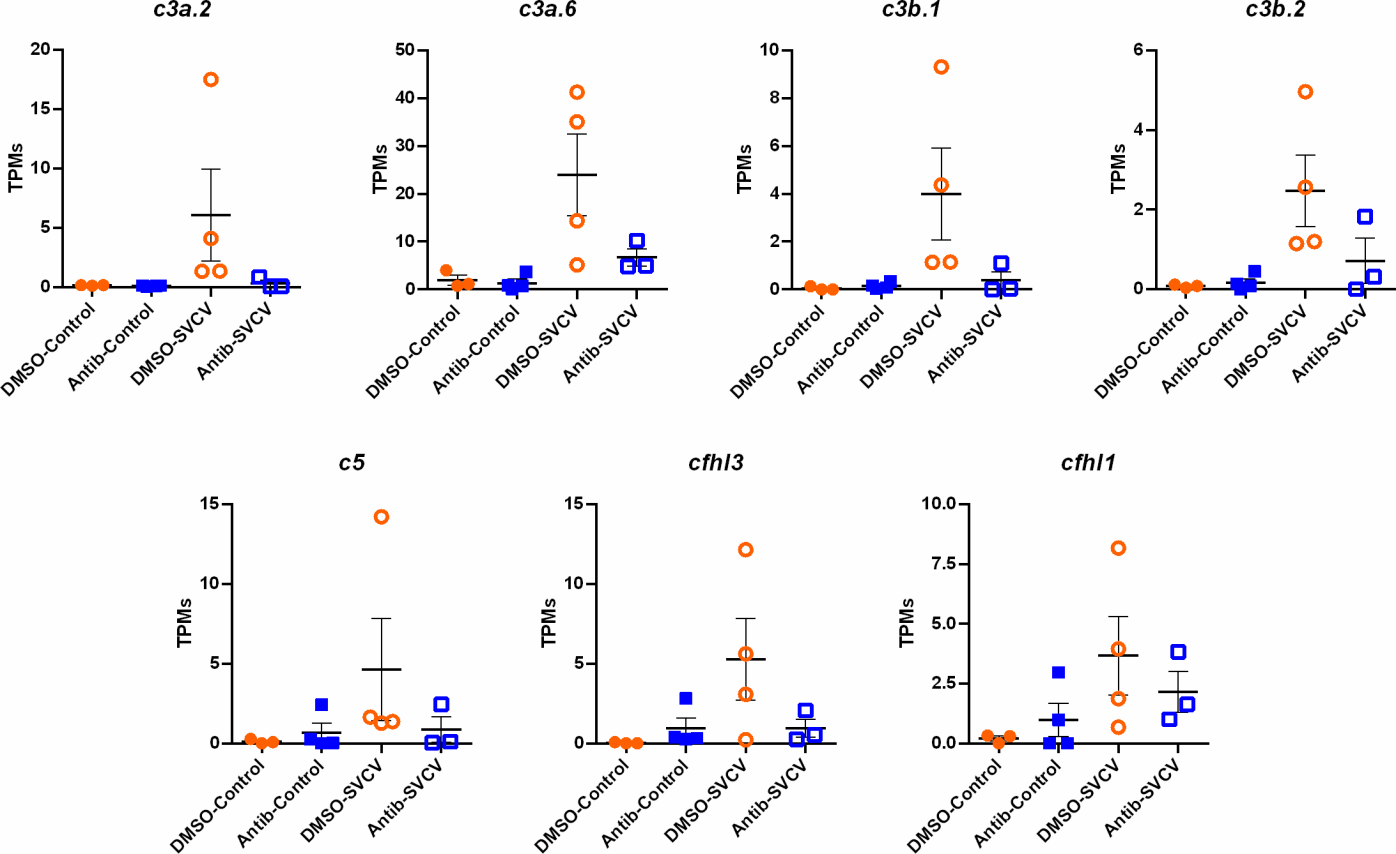
Representation of the TPM values of the complement genes significantly modulated in the kidneys of fish exposed to DMSO after challenge with SVCV (DMSO-SVCV) compared with the DMSO-control group.

For the intestine, a representation of the TPM values of the DEGs involved in the complement pathway, blood coagulation and haemoglobin synthesis is shown in **Figure 8**. Although the level of these genes was generally higher in the animals exposed to DMSO compared to those exposed to antibiotics, independent of the infection, some of them were downregulated by SVCV challenge in the DMSO-treated fish (*c4*, *serpina1*, *serpina1l*, *serpinf2a*, *tfa*), whereas they were not significantly affected by the infection in the fish exposed to antibiotics (**Figure 8**). Contrary to that observed in the intestine, certain complement genes were upregulated in the kidney after SVCV infection in DMSO-exposed fish, but this response was completely repressed in the animals treated with SMX-CLA (**Figure 9**). In the case of this tissue, a significant effect on blood coagulation and haemoglobin synthesis-related genes was not observed as a consequence of the antibiotic treatment and/or SVCV infection.

To confirm these results, we conducted qPCR analysis of some complement-, coagulation- and haemoglobin synthesis-related genes inhibited in the intestine after antibiotic exposure using a different batch of samples than that used for the RNA-Seq analysis. We confirmed that all the tested genes showed a lower expression in the intestine of animals exposed to SMX-CLA than in those treated with the vehicle alone, and statistically significant downregulation was even observed for some genes (*c1s*, *c3a.1*, *c5*, *c8a*, *c9*, *cp*, *tfa*) (**Supplementary Figure S4**).

### 3.6 Antibiotic exposure significantly reduces the number of macrophages and neutrophils in zebrafish

**Figure 10:**
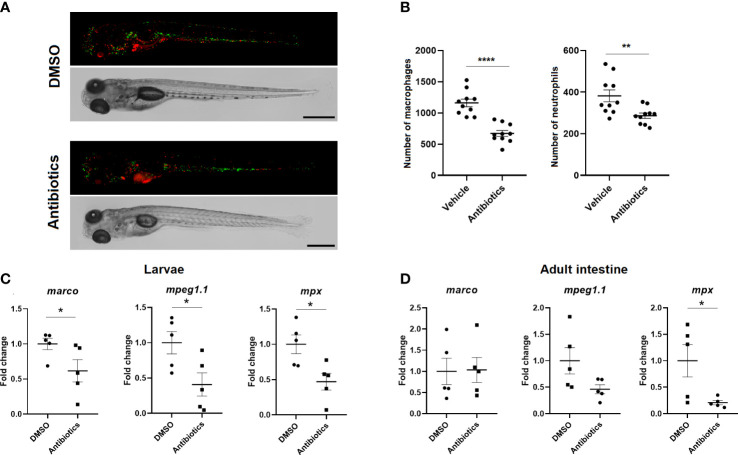
Effect of long-term exposure to SMX+CLA on the number of innate immune cells. **(A)** Representative images of double transgenic *Tg(mpeg:mCherry/mpx:GFP)* zebrafish larvae exposed to vehicle alone (DMSO) or SMX+CLA for 7 days. Scale bar: 500 µm. **(B)** Macrophage and neutrophil counts in DMSO- and SMX+CLA-treated larvae. Statistically significant differences are displayed as **** (p<0.0001) and ** (p<0.01). **(C)** Expression level of the macrophage (*marco*, *mpeg1.1*) and neutrophil (*mpx*) gene cell markers in DMSO- and SMX+CLA-treated larvae for 7 days and **(D)** in the intestine of adult zebrafish exposed to the treatments for 2 weeks. Statistically significant differences are displayed as * (p<0.05).

Double-transgenic *Tg(mpeg:mCherry/mpx:GFP)* larvae exposed for 7 days to DMSO or SMX-CLA were analysed by fluorescence microscopy to determine whether exposure to antibiotics has an effect on the total number of macrophages and neutrophils. The results reveal that SMX+CLA significantly reduced the number of both immune cell types compared with the larvae exposed to the vehicle alone (**Figure 10A, B**). qPCR analysis of the macrophage markers *marco* and *mpeg1.1* and the neutrophil marker *mpx* also revealed significantly lower transcription of these genes in the larvae exposed to antibiotics (**Figure 10C**). When these markers were analysed in the intestine and kidney of adult fish, we detected significantly lower levels of *mpx* and a tendency towards lower detection of *mpeg1.1* transcripts in the intestine (**Figure 10D**). However, the RNA-Seq results reveal a significant inhibition of mpeg1.1 and mpeg1.2 in the intestine of fish exposed to SMX+CLA. No differences were observed in the kidney between DMSO- and antibiotic-treated zebrafish (**Supplementary Figure S5**).

## 4 Discussion

Worldwide consumption of pharmaceuticals has increased dramatically during the last few decades. Consequently, the presence of pharmaceutically active compounds in different water bodies has also risen due to wastewater from hospitals, pharmaceutical manufacturers, households, livestock and aquaculture farms, among others (29). Antibiotics are among the most frequently detected pharmaceutical compounds in raw wastewater, together with analgesics, antibiotics, psychoactives, antihypertensives, anticholesteremics, and stimulants (30, 31). Unfortunately, wastewater treatment plants (WWTPs) are unable to completely remove pharmaceuticals due to their chemical and physical properties (31). As these compounds are active at very low concentrations, pharmaceutical pollution is an issue of special concern. The presence of antibiotics in drinking water has been reported in countries all around the world (32–39). Therefore, the impacts of antibiotics on human, animal and environmental health need to be analysed to better understand the consequences of this type of pollutant. Since sulfamethoxazole (SMX) and clarithromycin (CLA) are the most frequently observed antibiotics in European inland surface waters and are found at higher concentrations (15), these compounds were selected to determine the effect of an environmentally relevant concentration (0.01 mg/L each) on the microbiota and immune status of the model species zebrafish.

With regard to the intestinal and kidney microbiota, we observed that zebrafish exposed to SMX-CLA for 2 weeks did not show remarkable alterations in the microbiota composition in the kidney, although a higher total bacterial abundance was observed. On the other hand, an evident dysbiosis in the intestine was found in the antibiotic-treated fish compared to the controls. However, contrary to what could be expected, the bacterial alpha-diversity was not significantly affected, and a higher abundance of total bacteria was even observed in the intestine of fish exposed to antibiotics compared to the controls. The main classes of bacteria identified in the intestine of the zebrafish used in our study are also predominant in other metagenomics analyses of microbial communities in the intestine of zebrafish (40–45). Nevertheless, the zebrafish intestinal microbiota can show variations depending on the developmental stage, life history, diet and local environment (40, 41). In our metatranscriptome analysis, we found that the fish exposed to antibiotics showed a significant increase in the classes Chloroflexia and Alphaproteobacteria and a reduction in Actinomycetia, Bacilli and Gammaproteobacteria, with the variations in Alphaproteobacteria and Gammaproteobacteria being stronger. An increase in the relative abundance of Alphaproteobacteria in the intestines of zebrafish exposed to antibiotics was previously reported for oxytetracycline (OTC) (44). At the phylum level, this antibiotic reduced the abundance of Proteobacteria, although no differences were observed for SMX (43). For the mycobiota, DMSO-treated fish showed a higher abundance of Sordariomycetes and Saccharomycetes, followed by Eurotiomycetes, Dothideomycetes and Lecanoromycetes. Moreover, the diet and rearing environment both impact the intestinal fungal composition of zebrafish. Siriyappagouder et al. found that wild-caught-laboratory-kept (Uttara, India) zebrafish showed a predominance of Dothideomycetes, whereas their laboratory-reared counterparts (Bodø, Norway) showed a predominance of Saccharomycetes (46). Our analysis reveals that the fungi tended to show increased mean total abundance after antibiotic treatment but with a lower alpha diversity. The abundance of the class Sordariomycetes significantly increased after SMX+CLA exposure, but the classes Saccharomycetes, Dothideomycetes, Eurotiomycetes, Leotiomycetes and Exobasidiomycetes decreased in relative abundance. Taken together, we can conclude that zebrafish exposed to SMX+CLA at an environmentally relevant concentration for two weeks showed an evident alteration of their intestinal microbiota. In addition, the kidney was also affected to some extent, since a higher abundance of total bacteria was observed.

Although clinical exposure to antibiotics reduces bacterial abundance, subclinical exposures in the range of environmental concentrations of antibiotics can favour the proliferation of bacterial lineages with high genetic diversity and adaptive plasticity (14). The activation of the bacterial SOS response induced by low levels of antibiotics can result in an increased mutation rate and higher horizontal transfer of genetic material between bacteria, which in turn increases the phenotypic variability (14). This is in agreement with our results, where a higher raw abundance of bacteria was observed in fish exposed to SMX+CLA, which seems to be due to the proliferation of certain classes. Certain bacteria also produce metabolites, such as lactic acid, butyrate or tryptophan-derived metabolites (aryl hydrocarbon receptor ligands), that inhibit or restrict the colonization and/or growth of certain fungi (47). Therefore, alterations in the bacterial community composition impact the mycobiota composition and abundance, as was observed in this work.

Currently, it is widely known that the microbiome plays a pivotal role in the development and function of the host’s immune system and that the immune system controls host-microbe symbiosis. Therefore, the microbiota and immunity support intricate bidirectional communication (48). Different microbial components, such as bacterial lipopolysaccharide (LPS), flagellin, peptidoglycan or lipoteichoic acid (48), and fungal cell wall constituents, such as β-glucans and chitin (47), interact with host immune receptors (known as pattern recognition receptors -PRRs-) and elicit a variety of immune effects in host cells (47, 48). At the same time, bacteria and fungi are producers of a multitude of secondary metabolites with the ability to translocate from the intestinal lumen to different organs or tissues through the circulatory system and elicit tissue-specific immune responses with anti- or promicrobial effects (48). It is well known that microbial dysbiosis is involved in a multitude of immunological diseases (49) and can also increase the susceptibility to opportunistic bacteria or fungi as well as viral pathogens (50).

It has been previously reported that fish exposed to antibiotics are more susceptible to certain infectious diseases. This is the case for adult zebrafish exposed to SMX and OTC, which were more susceptible to the bacterial pathogen *Aeromonas hydrophila* and showed several immune parameters (alkaline phosphatase activity, expression of cytokines and antioxidant activity) affected in the intestine (43). In this work, we observed that zebrafish exposed to SMX+CLA were more susceptible to SVCV. Even a sublethal dose of SVCV induced a certain mortality in the antibiotic-treated fish. Numerous studies have suggested that a normal gastrointestinal microbiota is required for a normal antiviral response and that dysbiosis has an impact on antiviral immunity (50). A healthy microbiota helps maintain an optimal mucosal barrier, contains bacteria able to secrete virucidal antimicrobial peptides, inhibits viral attachment to host cells and modulates antiviral innate and adaptive immune processes (50). Mice orally treated with an array of antibiotics responded to viral infections with an impaired antiviral immune response and lower viral clearance, which resulted in more host damage and a higher mortality rate (51–54). Moreover, maternal antibiotic treatment altered the normal development of the neonatal microbiota and impacted the antiviral immunity of the progeny (55). Nevertheless, certain immune-suppressive activities mediated by antibiotics could be due to a toxic direct effect of the antibiotics, since it has been shown that fish treated with antibiotics through medicated feeds and bath treatments at clinical dosages can exhibit hepatotoxicity and several histopathological effects in different tissues, mainly as a consequence of an increase in oxidative stress (56).

The transcriptome analysis of zebrafish exposed to environmental concentrations of SMX+CLA revealed a significant alteration of the transcription of several immune genes in the intestine, with a remarkable inhibition of a multitude of genes belonging to the complement system and its closely related pathway, blood coagulation. Impaired immune functions and increased inflammation were previously reported in other fish species exposed to clinical or environmental concentrations of antibiotics (revised in 56). In addition, alterations or certain complement parameters were previously reported in different fish species exposed to different antibiotics. Common carp (*Cyprinus carpio*) exposed to environmental concentrations of metronidazole for 30 days showed a decline in complement activity, among other affected immune functions (57). Zebrafish larvae exposed to six types of β-diketone antibiotics from 6 dpf to 30 dpf showed lower detection of the complement component C3 (58). Guardiola et al. evaluated the complement activity and *c3* gene expression level in gilthead sea bream (*Sparus aurata*) fed OTC; among other enzymatic activities and immune genes, they found that the complement activity was significantly lower in the serum and that c3 expression was lower in the gut of OTC-treated fish (59). Contrary to those observations, some publications reported an increase in the complement component contents. He et al. found that hybrid tilapia fed florfenicol showed a higher quantity of the complement components C3 and C4 (60). Moreover, an acute oral administration of neomycin to crucian carp (*Carassius auratus gibelio*) revealed increases in the complement C3 content in the blood (61). Interestingly, although we did not observe an inhibition of the complement and coagulation components in the kidney of fish exposed to SMX-CLA in the absence of infection, those animals showed a reduced ability to increase the transcription of the complement components after the SVCV challenge. These results seem to indicate that the main immune and haematopoietic tissue in fish, the kidney, has a limited response to pathogens, which could explain the higher mortalities in response to SVCV.

Since it has been described that antibiotics can alter the leukocyte counts in fish, usually by decreasing their numbers, although with exceptions (62), we wanted to elucidate whether the SMX+CLA concentrations used in this experiment could impact the number of immune cells. In recent years, it has been shown that the intestinal microbiota plays a pivotal role in the regulation of haematopoiesis (63–67), and gut dysbiosis has been found to be associated with haematological abnormalities in both humans and mice (revised in 68). Moreover, decreases in the number of immune cells could explain the lower transcription of complement genes. We found that zebrafish larvae exposed for 7 days to both antibiotics showed a lower number of macrophages and neutrophils, which was corroborated by the qPCR analysis of macrophage and neutrophil cell markers. On the other hand, when these markers were analysed in the intestine and kidney from adult zebrafish, only a significant reduction in the level of the neutrophil marker *mpx* was observed in the intestine, although *mpeg1.1* and *mpeg1.2* were found to be inhibited in the RNA-Seq analysis. Therefore, we cannot rule out the possibility that the lower transcription of the complement and coagulation genes in the intestine and the absence of induction of certain complement genes in the kidney after infection with SVCV in those fish exposed to antibiotics is due to a reduced number of immune cells. Nevertheless, a massive downregulation of immune genes was not observed after exposure to antibiotics, especially in the kidney, which could also indicate a modulation of *mpx*, *mpeg1.1* and *mpeg1.2* gene expression in the intestine without affecting the number of macrophages and neutrophils in adult zebrafish.

A direct relationship between the microbiome and the complement pathway has been reported. Chehoud et al. observed that conventionally raised mice showed a higher expression of a broad variety of complement components in the skin than their germ-free mouse counterparts (69). However, although haematopoiesis was not evaluated in that work, the authors found that the inhibition of a key component of the complement system, the complement component C5a receptor (C5aR), resulted in an altered composition and diversity of the skin microbiota, reduced cell infiltration and inhibited the transcription of immune genes in the skin. Complement and microbiota seem to have a bidirectional relationship where microbiota alter the complement cascade and the complement alters the microbiota (69). A tight relationship between C5a/C5aR hyperactivation or repression and the gut microbiota has also been reported in mice (70). C3 KO mice also showed altered faecal microbiota, which could be involved in the frequent constipation phenotypes observed in these mice (71). Complement has also been linked to periodontal dysbiosis and inflammation (72). Therefore, we cannot rule out the possibility that the alterations of the complement system observed in the zebrafish exposed to SMX+CLA could be a consequence of the direct interplay between the microbiota and the complement.

In conclusion, our results reveal that zebrafish exposed to an environmentally relevant concentration of the two most frequently found antibiotics in European inland surface water, sulfamethoxazole (SMX) and clarithromycin (CLA), for two weeks showed a higher susceptibility to the viral pathogen SVCV than their vehicle-treated counterparts. Transcriptome analysis of the intestine and kidney revealed that SMX+CLA exposure significantly modulated several genes, especially in the intestine. Complement and blood coagulation, two intimately linked processes, were among the most affected processes, with a strong downregulation of several genes. These processes were not apparently affected in the kidney. Moreover, the transcriptome analysis of SMX+CLA-treated and control fish at 24 hpi with a sublethal dose of SVCV showed a completely different response in the experimental groups. The fish not previously exposed to antibiotics showed a more typical antiviral response, but the zebrafish exposed to SMX+CLA responded to the infection with an impaired immune response, characterized by an inability to overexpress the complement genes in the kidney. Metatrascriptome analysis revealed an altered microbiota in the intestine that, based on the literature, could explain the reduced complement pathway response. Additionally, this deficient complement response in the SMX+CLA-treated fish could also be a consequence of a reduced number of immune cells in the animals exposed to antibiotics. Since microbiota alterations have been linked to impaired haematopoiesis, the three factors (microbiome, haematopoiesis and complement signalling) could be interconnected and could explain the higher susceptibility of the zebrafish to viral infection after they were exposed to SMX+CLA. Much more research is needed to fully understand the immunosuppressive effects of antibiotics as environmental pollutants.

## Data availability statement

The datasets presented in this study can be found in online repositories. The names of the repository/repositories and accession number(s) can be found here: PRJNA893568 (SRA).

## Ethics statement

The animal study was reviewed and approved by CSIC National Committee on Bioethics, approval number ES360570202001/21/FUN.01/INM06/BNG01.

## Author contributions

PP: Methodology, bioinformatic analysis, validation; writing - original draft. MR-C: Bioinformatic analysis, writing - review & editing. AF: Bioinformatic analysis, writing - review & editing. BN: Conceptualization, funding acquisition, supervision; writing - review & editing. All authors contributed to the article and approved the submitted version.
