# Supplementary material for: An environmentally relevant concentration of antibiotics impairs the immune system of zebrafish (Danio rerio) and increases susceptibility to virus infection
Source: Front Immunol. 2023 Jan 12;13:1100092. doi: 10.3389/fimmu.2022.1100092 (PMC9878320; doi:10.3389/fimmu.2022.1100092)
Supplement: Supplementary file 5 [file DataSheet_5.docx]

Supplementary Material

# Supplementary Tables

# Table S1. Primer pairs used in this work.

| **Gene** | **Primer Forward** | **Primer Reverse** |
| --- | --- | --- |
| *c1s* | CTGGCGGTTGTCTCTCTGTT | GGGTCCTGACACTCATCGAC |
| *c3a.1* | GACGCCCAACTTGAAACCAC | TGTGACGACCTTCCAGATGC |
| *c4* | GGAACAGGACGGGGAACATT | CCAGCTCTCCCTTCACTGTG |
| *c5* | TCTGTCACGATGAGGGCAAC | GGACCCTGACTCCAAGAAGC |
| *c8a* | CACGAACAAGGAGATGGGCT | TCGGGCAGATCAGAAAGAGC |
| *c9* | GAGGTTGCCAGTCCCAATGA | ATACCAAATCCAGGAGCGGC |
| *masp1* | CGGTCCTGAATCTCCTACGC | ACAGGCACACGGTACACATC |
| *f5* | GGCAGGCGATGGATGAAGTA | CGAAATCACAACCCAGCAGC |
| *cp* | AACCCTCCCAAAGCACTGAC | CCACCTCATTTCCCAGTCCC |
| *tfa* | TGCTCCGTCCAATCATTGCA | ATTCCAGCCTCCAGACCTCT |
| *plg* | AATGAGTGCTGCCCTCTACG | AAAGTTCCCTCCCATCGCTG |
| *18s* | ACCACCCACAGAATCGAGAAA | GCCTGCGGCTTAATTTGACT |
| 16S rRNA (PSL-PSR) | AGGATTAGATACCCTGGTAGTCCA | ACTTAACCCAACATCTCACGACAC |

# Table S2. Summary of the Illumina sequencing, trimming and mapping against the zebrafish genome and fungal and bacterial databases.

| **Sample** | **Raw reads** | **Reads after trimming** | **Read length after trimming (bp)** | **Reads mapped to zebrafish (%)** | **Reads mapped to bacteria** | **Reads mapped to fungi** |
| --- | --- | --- | --- | --- | --- | --- |
| **DMSO_CINT3** | 20,420,008 | 20,419,843 | 146.47 | 97.92 | 585 | 543 |
| **DMSO_CINT4** | 20,291,236 | 20,291,027 | 146.21 | 96.5 | 270 | 658 |
| **DMSO_CINT5** | 20,281,002 | 20,280,853 | 145.42 | 97.25 | 965 | 914 |
| **ANTIB_CINT1** | 20,417,066 | 20,416,909 | 145.34 | 97.98 | 1057 | 10582 |
| **ANTIB_CINT2** | 20,317,680 | 20,317,508 | 144.49 | 95.9 | 11527 | 1129 |
| **ANTIB_CINT4** | 20,203,840 | 20,203,591 | 145.72 | 98.31 | 1726 | 6579 |
| **ANTIB_CINT5** | 20,369,178 | 20,368,977 | 143.87 | 96.06 | 5734 | 15177 |
| **DMSO_SVCVINT2** | 20,355,382 | 20,355,217 | 143.74 | 95.89 | 2800 | 3304 |
| **DMSO_SVCVINT3** | 20,292,864 | 20,292,673 | 144.38 | 95.49 | 2428 | 2023 |
| **DMSO_SVCVINT4** | 20,289,282 | 20,289,089 | 144.48 | 96.89 | 1409 | 2149 |
| **DMSO_SVCVINT5** | 20,249,118 | 20,248,934 | 144.5 | 97.87 | 635 | 1360 |
| **ANTIB_SVCVINT1** | 20,414,344 | 20,414,147 | 145.48 | 98 | 28566 | 1615 |
| **ANTIB_SVCVINT2** | 20,198,772 | 20,198,489 | 144.53 | 95.97 | 1004 | 18137 |
| **ANTIB_SVCVINT5** | 20,386,160 | 20,385,964 | 145.55 | 96.86 | 26279 | 1068 |
| **DMSO_CHK5** | 20,234,324 | 20,234,014 | 144.79 | 97.06 | 2274 | 3641 |
| **DMSO_CHK3** | 20,288,354 | 20,288,108 | 143.62 | 96.99 | 2480 | 3137 |
| **DMSO_CHK4** | 20,269,796 | 20,269,554 | 145.2 | 96.33 | 115 | 807 |
| **ANTIB_CHK5** | 20,400,340 | 20,400,177 | 144.05 | 97.68 | 479 | 602 |
| **ANTIB_CHK1** | 20,256,884 | 20,256,679 | 146.05 | 97.07 | 6474 | 18889 |
| **ANTIB_CHK2** | 17,798,098 | 17,791,864 | 142.27 | 96.71 | 1910 | 3385 |
| **ANTIB_CHK4** | 20,180,534 | 20,180,329 | 144.73 | 97.06 | 207 | 14049 |
| **DMSO_SVCVHK5** | 20,265,686 | 20,265,470 | 144.33 | 96.78 | 2202 | 3317 |
| **DMSO_SVCVHK2** | 20,350,004 | 20,349,806 | 143.56 | 95.26 | 4649 | 6436 |
| **DMSO_SVCVHK3** | 20,227,342 | 20,227,127 | 143.52 | 94.51 | 833 | 1392 |
| **DMSO_SVCVHK4** | 20,193,004 | 20,192,819 | 145.58 | 96.96 | 1392 | 2287 |
| **ANTIB_SVCVHK5** | 20,222,738 | 20,222,535 | 146.23 | 96.87 | 12306 | 2838 |
| **ANTIB_SVCVHK1** | 20,150,430 | 20,150,212 | 144.74 | 96.91 | 2312 | 1227 |
| **ANTIB_SVCVHK2** | 20,214,502 | 20,214,270 | 142.87 | 96.98 | 9187 | 7837 |

#
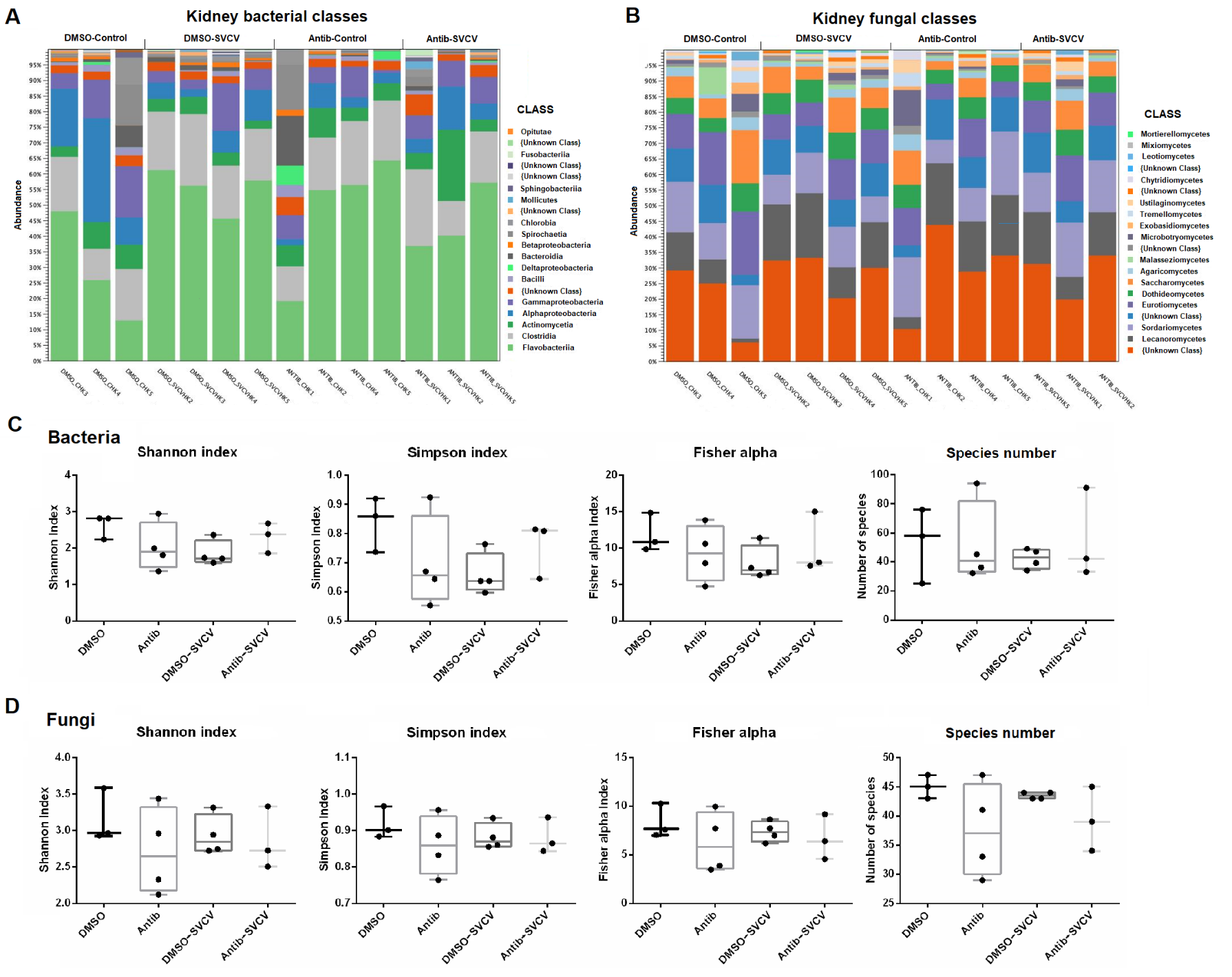
Supplementary Figures

**Supplementary Figure 1.** **Taxa relative abundances in the kidneys of zebrafish treated with DMSO (vehicle) or SMX+CLA under SVCV-infected and uninfected conditions.** **A)** Relative abundance of the main bacterial classes. **B)** Relative abundance of the main fungal classes. **C, D)** Shannon entropy, Simpson, Fisher alpha and species number indices of **C)** bacteria and **D)** fungi in the kidney of the experimental groups.

**
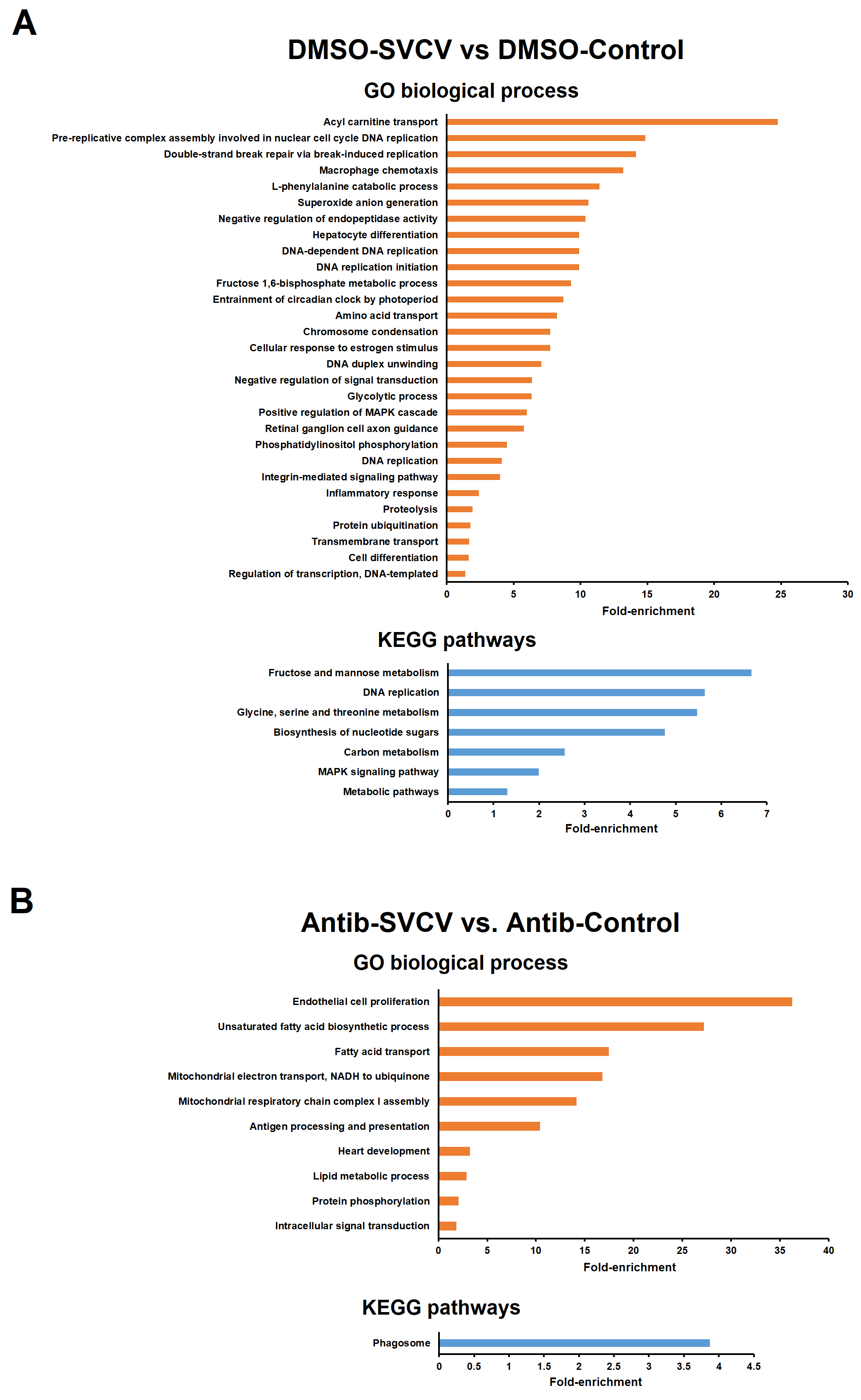
**

**Figure S2. GO and KEGG pathway enrichment analyses of the genes significantly modulated in the intestine of the A) DMSO- or B) antibiotic-treated fish after SVCV infection.**

**
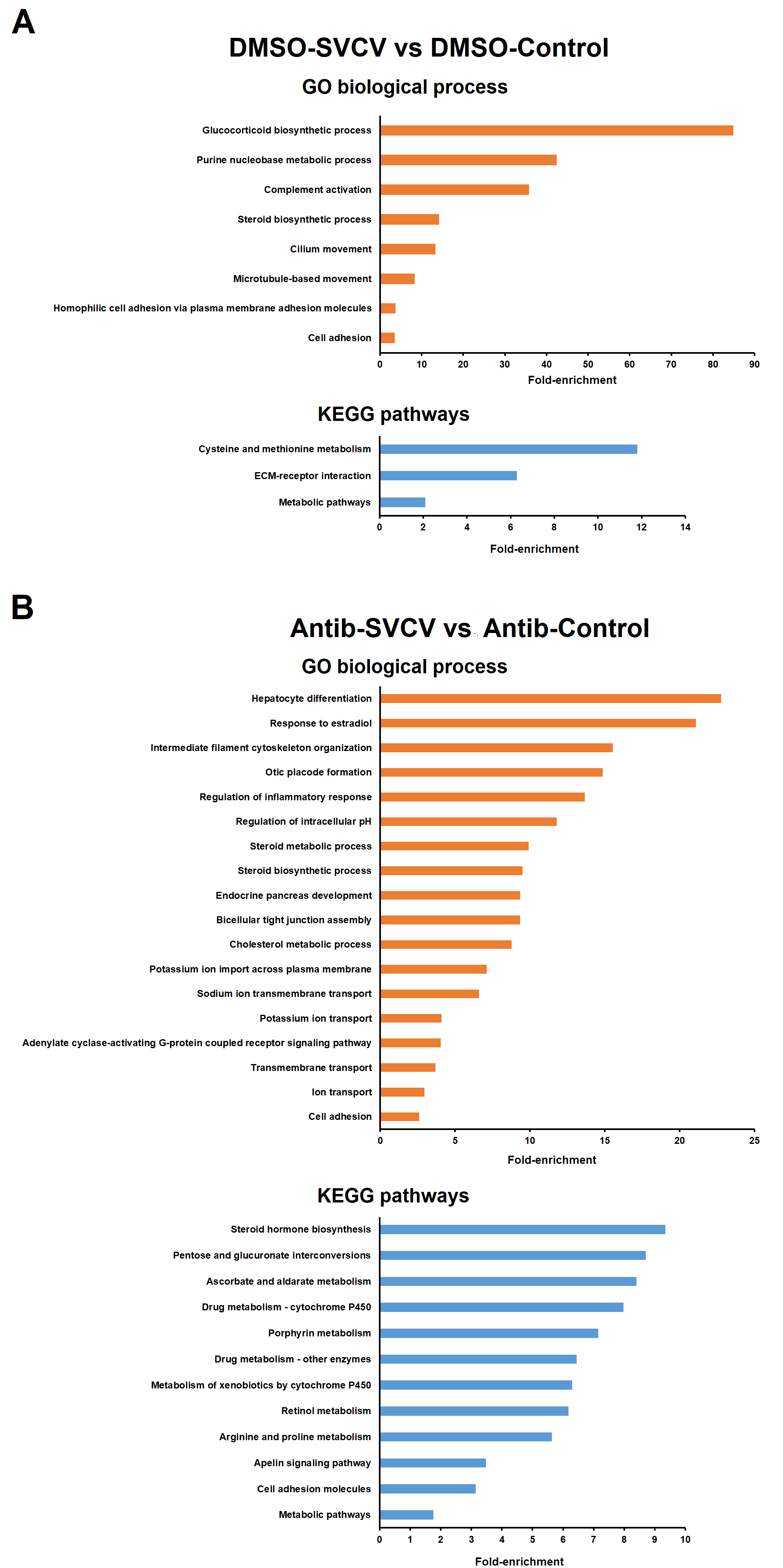
**

**Figure S3. GO and KEGG pathway enrichment analyses of the genes significantly modulated in the kidney of the A) DMSO- or B) antibiotic-treated fish after SVCV infection.**

**
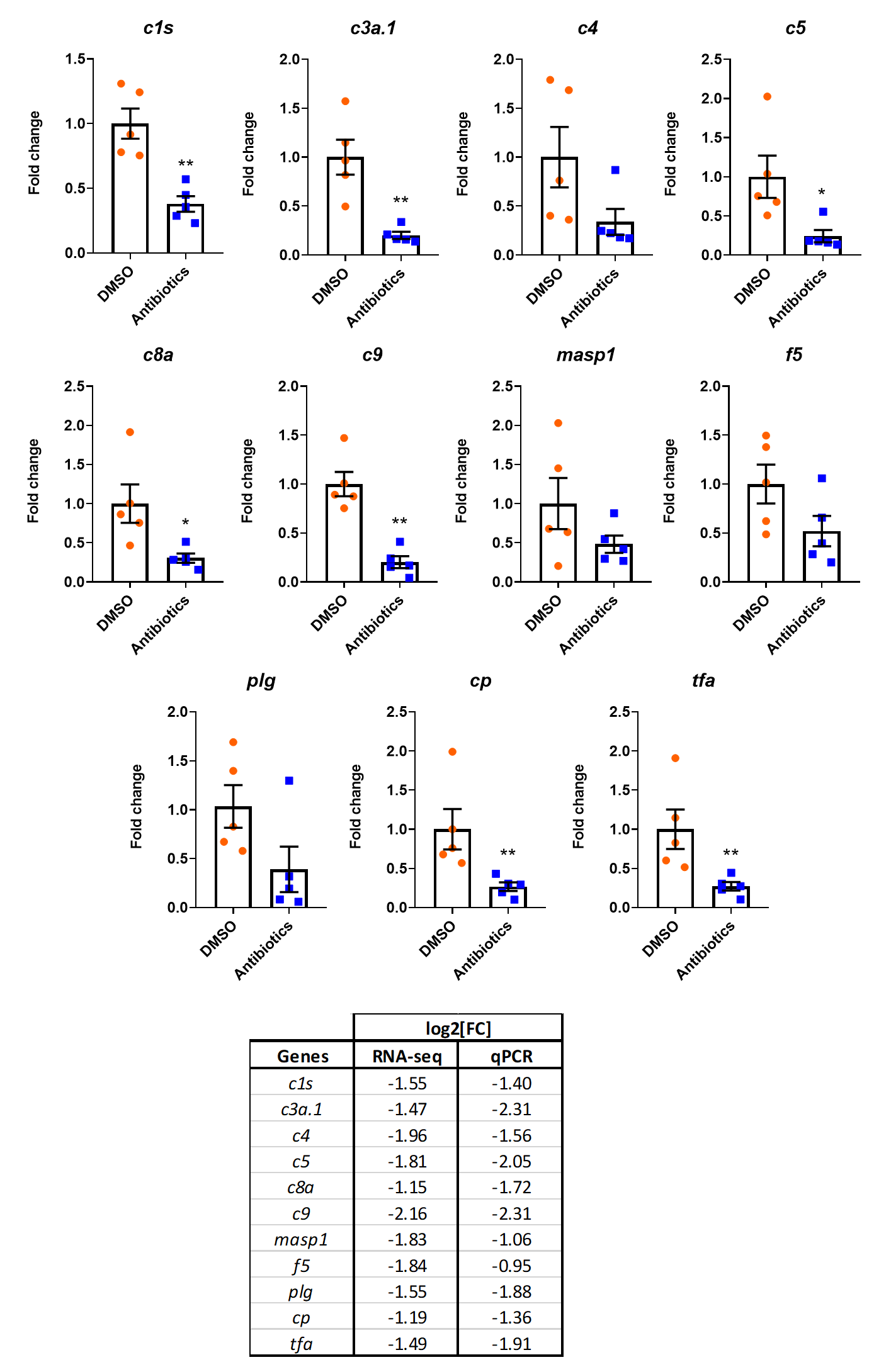
**

**Figure S4. qPCR validation of the RNA-Seq results.** Eleven genes related to the complement and/or coagulation pathways and showing lower expression in the intestine of SMX+CLA-treated fish than the control fish were analysed by qPCR. This analysis confirmed the expression pattern observed in the RNA-Seq results. Statistically significant differences are displayed as ** (p<0.01), * (p<0.05).

**
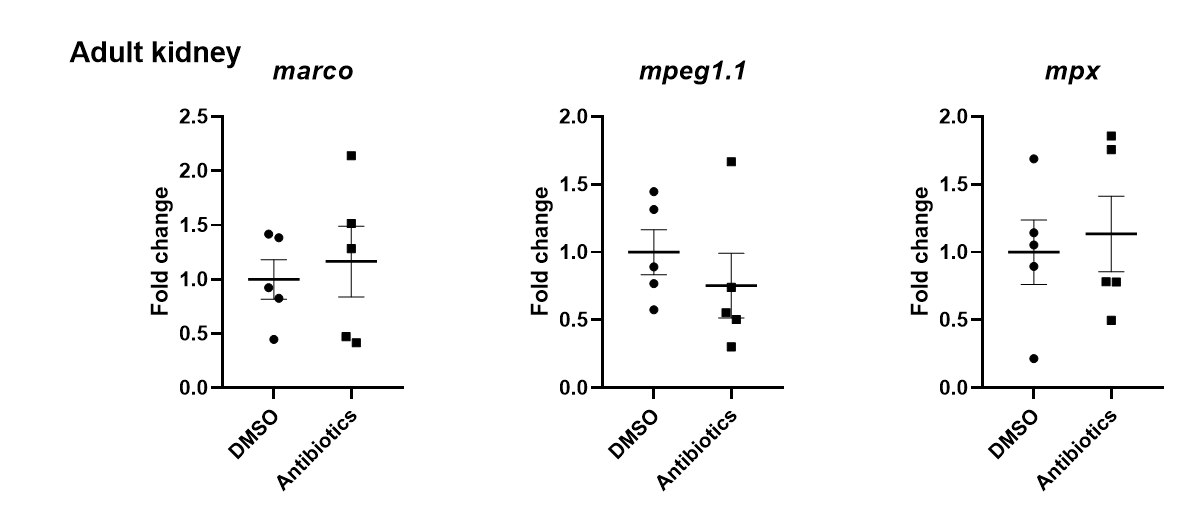
**

**Figure S5. Effect of long-term exposure to SMX+CLA on the expression levels of** macrophage (*marco*, *mpeg1.1*) and neutrophil (*mpx*) gene cell markers in the kidney of adult zebrafish exposed to the treatments for 2 weeks. No statistically significant differences were observed.
